# Assessment of selection bias due to dropouts in the follow-up of the Scania Public Health Cohort

**DOI:** 10.1177/1403494820919544

**Published:** 2020-05-28

**Authors:** Catarina Canivet, Anton Nilsson, Jonas Björk, Mahnaz Moghaddassi, Per-Olof Östergren

**Affiliations:** 1 Social Medicine and Global Health, Department of Clinical Sciences in Malmö, Lund University, Sweden; 2 EPI@LUND (Epidemiology, Population studies and Infrastructures at Lund University), Department of Laboratory Medicine, Lund University, Sweden; 3 Clinical Studies Sweden, Forum South, Skåne University Hospital, Sweden

**Keywords:** Population-based study, register data, bias, selection, epidemiology, follow-up studies, cohort studies, public health

## Abstract

*Aims:* To investigate potential differences between participants and dropouts in the 2005 follow-up of the Scania Public Health Cohort Study regarding the prevalence of commonly studied health determinants and whether these factors had differential associations with three health outcomes: all-cause mortality and purchase of prescribed cardiovascular or psychotropic drugs during a 10-year follow-up period. *Methods:* The Scania Public Health Cohort was initiated in 1999/2000, with randomly invited participants aged 18–80 years from the general population (58% participation). Questionnaire data from 10,462 participants and 2576 dropouts in the 2005 follow-up (80% participation) were linked to public registers on mortality and purchase of prescribed drugs. *Results:* Age, male gender, being born abroad, low educational level, low self-rated mental and general health and daily smoking were all related to dropping out. The 10-year mortality was higher among dropouts (13.4% versus 11.9%; age-adjusted hazard ratio 1.6, 95% confidence interval: 1.4–1.8). In 13 out of 18 analyses, similar associations between health determinants and outcomes were found across participants and dropouts. However, being born outside of Sweden was associated with higher risks for all three poor health outcomes among participants, but not so among dropouts. ***Conclusions:* Despite selective participation at follow-up, there was little evidence of selection bias, insofar as estimated associations were generally similar across participants, dropouts and the whole cohort. This finding is important for the assessment of the validity of prospective findings from this cohort and similar ones, where the loss of individuals at consecutive follow-ups of exposure is non-negligible.**

## Introduction

Prospective cohort studies performed during the past few decades have delivered invaluable information about causes of disease, of which some are at least partly preventable [1, 2]. However, participation in such studies is steadily declining throughout the Western world, from around 80% previously to 30–40% or below nowadays [3]. Moreover, certain groups are generally under-represented, such as men, persons with low education and/or socioeconomic status and those already in poor health [4]. This could undermine the validity of findings because of selection bias, namely, the estimated effects among participants differ systematically from what would had been obtained in the target population as a whole, if data on everyone were available.

Selection bias will generally occur when the selection of individuals under study depends on at least two factors: the exposure under study (or a cause of the exposure) and the outcome (or a cause of the outcome) [5]. Because the factors that determine self-selection into cohort studies may often belong to these categories, selection bias is clearly a concern. The tendency to drop out from consecutive follow-ups has been considered an even greater problem than initial non-participation, because the choice to continue in the study may be influenced by ongoing experience of the outcome in question [6]. Selection bias can also occur if the effect of an exposure (e.g. alcohol consumption) is heterogeneous across levels of a health determinant (e.g. socioeconomic status) that is related to study participation [7, 8]. Self-selection of healthier individuals at recruitment and follow-up may also considerably hamper the possibility of studying exposures, combinations of risk factors or subgroups that are poorly represented in the study sample [9].

Nevertheless, several previous studies have found that neither initial non-participation nor dropping out at consecutive follow-up created any substantial bias in risk estimates [10–15]. For example in the Danish National Birth Cohort, with only 30% participation, the bias was estimated to be less than 16% for the examined exposure-outcome associations (in vitro fertilization and preterm birth, smoking and small-for-gestational age infant, and body mass index and stillbirth) [16]. However, an investigation of the effect of dropping out in the same study revealed an underestimation of the relationship between socioeconomic inequalities and several outcomes, which increased over time [17]. Although similar results were found in data from the Avon Longitudinal Study of Parent and Children study, the authors concluded that qualitative inferences about the direction and approximate magnitude of the inequalities were still valid [18].

The Scania Public Health Cohort (SPHC) is an ongoing general population study. Questionnaires concerning sociodemographic data and a large number of psychosocial and health-related variables were sent out by mail at baseline in 1999/2000, and thereafter in 2005, 2010 and 2016. A number of reports from this cohort have also included registry information [19, 20]. The study design has been described previously [21], and the representativity of the baseline participants has been assessed by comparing age, gender, educational level, country of birth and healthcare expenditure with information from a register covering the entire adult county population of over 850,000 individuals. Immigrants, especially from non-Nordic countries, were the only group notably under-represented and the survey sample had about the same healthcare utilization costs as the general population [21]. Two decades have now passed since the initiation of the study and the issue of selection bias needs to be re-visited. An investigation of selection bias in the SPHC study may also contribute to an understanding of the general magnitude of this problem in cohort studies.

## Aims

One aim of the present study was to investigate whether there was selective participation in the first follow-up in 2005 of the SPHC as reflected in differences between participants and non-participants regarding age, gender, country of origin, education, baseline self-rated health and self-rated mental health and smoking, and further, whether a selection of healthy participating individuals took place, as seen in subsequent mortality and use of medicines for cardiovascular disease and mental health problems during a 10-year follow-up. Moreover, and more importantly, the study aimed to investigate whether the determinants assessed at baseline had differential associations with the outcomes (mortality and use of medicine as described above) across participants, non-participants and the whole cohort, that is, whether there were indications that a significant selection bias was present, which should have to be acknowledged in future studies from this cohort.

## Methods

### Participants

The design of the SPHC, initiated in 1999/2000, has been described elsewhere [21]. In short, 23,437 individuals between 18 and 80 years of age from the general population in the region of Scania (population 1.3 million) in southern Sweden were randomly invited to respond to a mailed questionnaire containing more than 120 questions about sociodemographic background factors, living and working conditions, health behaviours and self-rated health. The response proportion was 58% (*N*=13,589). All respondents in the baseline survey who were still alive and residing in Scania were invited to each of the three consecutive follow-ups in 2005, 2010 and 2016; each follow-up used a very similar questionnaire. The study was approved by the Regional Ethical Review Board at Lund University, Sweden (1999/99, 2005/471, 2010/392, 2015/471 and 2016/622).

We examined the relationships between several health determinants and participation in the 2005 follow-up. Age, gender, country of birth and educational level were selected because they all constitute part of the standard sociodemographic background factors for most epidemiological studies and are important for assessing gender equity and other health equity issues. We also selected two measures of self-assessed health (general and mental health), because we wanted to investigate whether health is an important factor for continued participation in cohort studies based on general populations. Finally, we also selected the variable daily smoking, because it represents one of the most important health determinants and often plays the role of exposure, outcome or potential confounder in epidemiological studies.

Age and gender were obtained from the population register used for recruiting the invited individuals. Country of origin was recorded as ‘born in Sweden’ or ‘not born in Sweden’. Educational level at baseline was determined by the self-reported total years of formal education and dichotomized as ⩽ 12 years versus ⩾ 13 years. Mental health was assessed with the 12-item version of the General Health Questionnaire (GHQ-12). We used the 0-0-1-1 scoring method (range 0–12) with poor mental health (‘GHQ-caseness’) defined as a score of 2 or higher [22, 23]. Self-rated health was measured with the question ‘In general, how do you rate your current health status?’ with five response alternatives, ranging from ‘very good’ to ‘very poor’ [24]. The answers were dichotomized as good (the first two alternatives) versus poor (the other three). Smoking habits were dichotomized as ‘smoking daily’, yes/no.

From baseline in 1999/2000 and until the first follow-up in 2005, the mortality was 4.1%. Age-adjusted mortality hazard ratios (HR) during this period were for men versus women 1.5 (95% confidence interval (CI) 1.2–1.7), for persons born abroad versus those born in Sweden 1.4 (1.1–1.9), for those with an educational level of ⩽ 12 years versus ⩾ 13 years 1.1 (0.9–1.4), for poor self-rated mental health 1.7 (1.4–2.1), for poor self-rated health 2.0 (1.7–2.3) and for daily smoking 2.5 (2.0–3.0) (data not shown in tables).

This study presents results concerning those, out of the original cohort, who were alive at 31 December 2005 (*N*=13,038), out of whom 10,462 (80%) had participated in the 2005 follow-up and out of whom 2576 had dropped out.

### Outcome variables

We considered three different outcome variables: all-cause mortality and purchase of two types of prescribed pharmaceuticals during follow-up; drugs for cardiovascular disease and drugs for mental health problems. Information on mortality during the period 2000–2015 has been added from the Cause of Death Register, as have data on pharmaceuticals purchased on prescription at pharmacies from the Swedish Prescribed Drug Register, covering the period 2005–2015. In Sweden, the system with unique personal identification numbers, provided by the Swedish National Tax Agency, permits linkage with these comprehensive registers. The researcher receives anonymised data after approved applications to the Regional Ethical Review Board, Statistics Sweden and the National Board of Health and Welfare. In the present study we report purchase (yes/no) from 2006 through 2015 of any prescribed drug pertaining to cardiovascular conditions, namely Anatomical Therapeutic Chemical (ATC) codes B01AA03 (vitamin K antagonists), B01AC (platelet aggregation inhibitors, excluding heparin), B01AE (direct thrombin inhibitors), B01AF (direct factor Xa inhibitors), C01A, C01B, C01D (cardiac therapy), C02 (antihypertensives), C03 (diuretics), C07 (beta-blocking agents), C08 (calcium channel blockers), C09 (agents acting on the renin-angiotensin system) and C10 (lipid modifying agents) and to psychiatric conditions, namely, ATC-codes N05A (neuroleptics), N05B (anxiolytics), N05C (hypnotics and sedatives) and N06A (antidepressants) [25].

### Statistical methods

**Figure 1.**
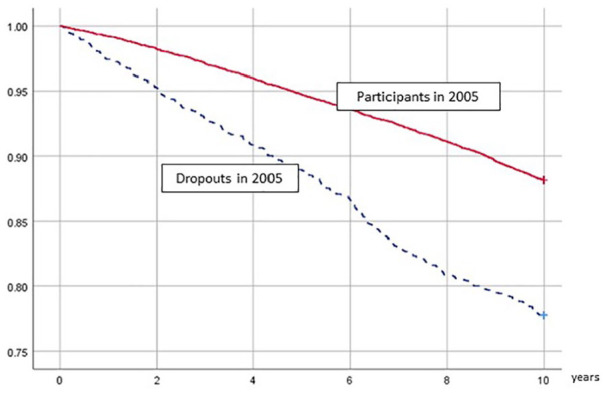
Survival in participants and dropouts, from 2006 through 2015, Scania Public Health Cohort. Dropouts are re-weighted with respect to age and gender to resemble participants.

The distributions of health determinants among participants and dropouts are presented as percentages and the relationships between these variables and participation in the 2005 follow-up were examined with logistic regression and expressed with both unadjusted and age-adjusted odds ratios (ORs) (Table I). An age-and gender-weighted Kaplan-Meier survival curve illustrates the mortality discrepancy between participants and dropouts (Figure 1).

**Table I.** Health determinants at baseline in 1999/2000 in relation to participation in the first follow-up (2005) of the Scania Public Cohort Study. Results are presented as numbers, frequencies (%) and OR for not participating. ORs are given as unadjusted and as age-adjusted, with 95% CI. *N*=13,038, that is, those of the original cohort (*N*=13,589) who were alive and still residing in Scania, by 31 December 2005.

Variables (missing data)	Total *N*	Participating in 2005 (*N* = 10,462)	Dropping out in 2005 (*N* = 2576)	OR for dropping out in 2005			
Age in 1999/2000		*N* / %	*N* / %	Unadjusted		Age-adjusted	
				OR	95% CI	OR	95% CI
18–30	2470	1698 / 16.2	772 / 30.0	1.8	1.5–2.1		
31–40	2367	1802 / 17.2	565 / 21.9	1.2	1.1–1.5		
41–50	2447	2017 / 19.3	430 / 16.7	0.8	0.7–1.0		
51–60	2609	2272 / 21.7	337 / 13.1	0.6	0.5–0.7		
61–70	1862	1649 / 15.8	213 / 8.3	0.5	0.4–0.6		
71–80	1283	1024 / 9.8	259 / 10.1	1			
Total	13,038	10462 / 100	2576 / 100				
Gender							
Female	7111	5809 / 55.5	1302 / 50.5	1		1	
Male	5927	4653 / 44.5	1274 / 49.5	1.2	1.1–1.3	1.2	1.1–1.3
Born in Sweden (155)							
Yes	11,513	9378 / 90.5	2135 / 84.6	1		1	
No	1370	980 / 9.5	390 / 15.4	1.7	1.5–2.0	1.7	1.5–1.9
Education level (596)							
⩾ 13 years	4598	3805 / 37.9	793 / 33.2	1		1	
⩽ 12 years	7844	6246 / 62.1	1598 / 66.8	1.2	1.1–1.3	1.4	1.3–1.5
Mental health (110)							
Good	9791	7976 / 76.8	1815 / 71.6	1		1	1
Poor	3137	2416 / 23.2	721 / 28.4	1.3	1.2–1.4	1.2	1.1–1.3
Self-rated health (99)							
Good	9135	7402 / 71.2	1733 / 68.1	1		1	
Poor	3804	2994 / 28.8	810 / 31.9	1.2	1.1–1.3	1.3	1.2–1.4
Daily smoking (209)							
No	10,497	8540 / 82.9	1957 / 77.6	1		1	
Yes	2332	1766 / 17.1	566 / 22.4	1.4	1.3–1.6	1.4	1.2–1.5

OR: odds ratio; CI: confidence interval.

Table IIa, Table IIb and Table III show HRs for the relationships between health determinants and outcomes in participants and dropouts, as determined by Cox regression. The same analyses were performed for the whole cohort (i.e. participants + dropouts), but as the results were nearly identical with those of the participants, they were omitted from Table IIa and Table IIb. In Table III, all factors were simultaneously adjusted for, and results for all three groups are presented.

**Table IIa.** Mortality from 2006 through 2015, by health determinants measured in 1999/2000 and by participatory status in 2005, Scania Public Health Cohort Study. *N* = 13038, of whom 10,462 participants and 2576 dropouts.

**Mortality**	Participants	Dropouts	Participants		Dropouts		Participants		Dropouts	
	Proportion deceased		Unadjusted HR				Age-adjusted HR			
	%	%	HR	95% CI	HR	95% CI	HR	95% CI	HR	95% CI
All	11.9	13.4	1		1.1	1.02–1.3	1		1.6	1.4–1.8
Gender										
Female	9.5	14.0	1		1		1		1	1
Male	14.8	12.7	1.6	1.4–1.8	0.9	0.7–1.1	1.7	1.5–1.9	1.4	1.1–1.7
Born in Sweden										
Yes	11.8	13.6	1		1		1		1	
No	11.4	9.7	1.0	0.8–1.2	0.7	0.5–0.99	1.3	1.1–1.6	1.0	0.7–1.4
Education level										
⩾ 13 years	6.3	5.7	1		1		1		1	
⩽ 12 years	14.2	14.2	2.3	2.0–2.7	2.6	1.9–3.6	1.1	0.96–1.3	1.3	0.97–1.9
Mental health										
Good	12.5	13.9	1		1		1		1	
Poor	9.3	11.1	0.7	0.6–0.8	0.8	0.6–1.02	1.3	1.1–1.5	1.3	1.02–1.7
Self-rated health										
Good	9.3	9.6	1		1		1		1	
Poor	18.1	21.2	2.0	1.8–2.3	2.4	1.9–3.0	1.6	1.4–1.8	1.3	1.01–1.6
Daily smoking										
No	11.3	12.9	1		1		1		1	
Yes	13.8	13.4	1.2	1.1–1.4	1.1	0.8–1.4	2.0	1.7–2.3	1.8	1.3–2.3

HR: hazard ratio; CI: confidence interval.

**Table IIb.** First-time purchase of any prescription-based cardiovascular or psychotropic medication from 2006 through 2015, by health determinants measured in 1999/2000 and by participatory status in 2005, Scania Public Health Cohort Study. *N* = 13,038, of whom 10,462 participants and 2576 dropouts.

**Cardiovascular medication**	Participants	Dropouts	Participants		Dropouts		Participants		Dropouts	
	Frequencies		Unadjusted HR				Age-adjusted HR			
	%	%	HR	95% CI	HR	95% CI	HR	95% CI	HR	95% CI
All	54.3	44.4	1		0.8	0.7–0.8	1		1.0	0.9–1.1
Gender										
Female	52.6	46.1	1		1		1		1	
Male	56.5	42.7	1.1	1.1–1.2	0.9	0.8–0.995	1.1	1.03–1.1	1.0	0.9–1.2
Born in Sweden										
Yes	54.2	44.3	1		1		1		1	
No	54.4	42.1	1.0	0.9–1.1	0.9	0.8–1.1	1.1	1.02–1.2	0.9	0.8–1.1
Education level										
⩾ 13 years	44.2	35.8	1		1		1		1	
⩽ 12 years	59.4	45.9	1.6	1.5–1.7	1.4	1.2–1.6	1.2	1.1–1.3	1.2	1.04–1.4
Mental health										
Good	55.5	44.7	1		1		1		1	
Poor	50.2	42.7	0.9	0.8–0.9	0.9	0.8–1.1	1.2	1.1–1.3	1.2	1.1–1.4
Self-rated health										
Good	49.3	37.7	1		1		1		1	
Poor	66.7	57.7	1.7	1.6–1.8	1.9	1.7–2.1	1.4	1.4–1.5	1.4	1.3–1.6
Daily smoking										
No	53.7	43.8	1		1		1		1	
Yes	56.2	44.9	1.0	0.96–1.1	1.0	0.9–1.2	1.1	1.1–1.2	1.0	0.9–1.2
**Psychotropic medication**										
All	43.8	42.7	1		1.0	0.9–1.1	1		1.2	1.1–1.3
Gender										
Female	50.1	51.4	1		1		1		1	
Male	35.8	33.9	0.6	0.6–0.7	0.6	0.5–0.6	0.6	0.6 (sic)	0.6	0.5–0.7
Born in Sweden										
Yes	43.1	42.1	1		1		1		1	
No	48.9	44.9	1.2	1.1–1.3	1.0	0.9–1.2	1.3	1.1–1.4	1.1	0.9–1.3
Education level										
⩾ 13 years	37.9	34.0	1		1		1		1	
⩽ 12 years	46.5	45.3	1.3	1.2–1.4	1.5	1.3–1.7	1.1	1.1–1.2	1.3	1.2–1.5
Mental health										
Good	40.7	38.6	1		1		1		1	
Poor	53.8	52.1	1.5	1.4–1.6	1.5	1.3–1.7	1.9	1.7–2.0	1.8	1.6–2.1
Self-rated health										
Good	36.9	34.4	1		1		1		1	
Poor	60.6	59.9	2.1	2.0–2.2	2.3	2.1–2.6	1.9	1.8–2.1	2.0	1.8–2.3
Daily smoking										
No	42.1	40.8	1		1		1		1	
Yes	52.0	48.8	1.3	1.2–1.4	1.3	1.1–1.4	1.4	1.3–1.5	1.3	1.1–1.5

HR: hazard ratio; CI: confidence interval.

**Table III.** Multivariate analysis of associations, separate for participants, dropouts and for the total Scania Public Health Cohort (*N*=13,038) in 2005, between health determinants measured in 1999/2000, and events (mortality, first-time purchase of any prescription-based cardiovascular medication and first-time purchase of any psychotropic medication) 2006–2015. All factors were entered simultaneously into the model and presented as age-adjusted HRs with 95% CIs.

	Participating in 2005 *(N*=10,462)		Dropping out in 2005 (*N*=2576)		Total cohort at follow-up in 2005 (*N*=13,038)	
Health outcome	HR	95% CI	HR	95% CI	HR	95% CI
**Mortality**						
Gender, male vs. female	1.8	1.6–2.1	1.4	1.1–1.8	1.7	1.6–1.9
Born in Sweden, no vs. yes	1.2	0.99–1.5	1.0	0.7–1.5	1.2	1.01–1.4
Education level at baseline, low vs. high	1.0	0.9–1.2	1.3	0.9–1.8	1.1	0.95–1.3
Mental health at baseline, poor vs. good	1.0	0.9–1.2	1.3	0.9–1.8	1.1	0.9–1.3
Self-rated health at baseline, poor vs. good	1.6	1.4–1.8	1.2	0.95–1.6	1.6	1.4–1.8
Daily smoking at baseline, yes vs. no	2.1	1.8–2.4	1.8	1.3–2.4	2.0	1.8–2.3
**Cardiovascular medication**						
Gender, male vs. female	1.1	1.1–1.2	1.1	0.98–1.3	1.1	1.1–1.2
Born in Sweden, no vs. yes	1.0	0.95–1.2	0.9	0.7–1.04	1.0	0.9–1.1
Education level at baseline, low vs. high	1.2	1.1–1.2	1.2	1.01–1.3	1.2	1.1–1.2
Mental health at baseline, poor vs. good	1.1	0.99–1.1	1.1	0.95–1.3	1.1	1.01–1.1
Self-rated health at baseline, poor vs. good	1.4	1.3–1.5	1.4	1.2–1.6	1.4	1.3–1.5
Daily smoking at baseline, yes vs. no	1.1	0.98–1.1	1.0	0.8–1.1	1.0	0.98–1.1
**Psychotropic medication**						
Gender, male vs. female	0.6	0.6–0.7	0.6	0.5–0.7	0.6	0.6–0.7
Born in Sweden, no vs. yes	1.2	1.1–1.3	1.0	0.8–1.2	1.1	1.03–1.2
Education level at baseline, low vs. high	1.1	1.004–1.1	1.3	1.2–1.5	1.1	1.1–1.2
Mental health at baseline, poor vs. good	1.4	1.3–1.6	1.4	1.2–1.6	1.4	1.4–1.5
Self-rated health at baseline, poor vs. good	1.7	1.6–1.8	1.7	1.5–2.0	1.7	1.6–1.8
Daily smoking at baseline, yes vs. no	1.2	1.2–1.3	1.2	1.03–1.4	1.2	1.2–1.3

HR: hazard ratio; CI: confidence interval.

## Results

As shown in Table I, age (younger and older, versus middle-aged), male gender, being born abroad, a low level of education, a baseline rating of one’s mental and general health as low and being a smoker (at baseline in 1999/2000) all increased the risk of dropout at the follow-up in 2005.

The age-adjusted HR for mortality from 2006 through 2015 was 1.6 (1.4–1.8) in dropouts versus participants (Table IIa). Age- and gender-weighted survival curves in the two groups are presented in Figure 1. Table IIa and Table IIb further show the associations between health determinants and poor health outcomes in participants and dropouts. In most analyses (13 out of 18 comparisons, i.e. 6 determinants × 3 outcomes), there was a similarity in associations across participants and dropouts, with estimated effects operating in the same direction. For example, daily smoking was a risk factor for mortality in both groups and poor self-rated health was a risk factor for purchase of prescribed cardiovascular medication in both groups. However, the HRs varied somewhat in magnitude, and particularly so for mortality. For instance, the age-adjusted HR for men was 1.7 (1.5–1.9) among participants, but only 1.4 (1.1–1.7) among dropouts. The HRs for the whole cohort were mostly identical or very close to those of participants (data not shown).

In five instances, estimated effects did not operate in the same directions for participants and non-participants. In particular, being born outside Sweden was associated with higher age-adjusted risks for all three poor health outcomes among participants, but not so among dropouts.

Table III shows, separately for participants, dropouts and the whole cohort, the multivariate analysis of associations between health determinants measured in 1999/2000 and the three poor health outcomes, with all health determinants simultaneously entered into the model. The discrepancy remains regarding the increased risk observed for foreign-born individuals among participants, but not for dropouts, for mortality as well as for purchase of prescribed psychotropic medication. Moreover, poor self-rated health at baseline stands out as a strong risk factor for mortality in participants (HR 1.6; 1.4–1.8), but to a lesser degree in dropouts (HR 1.2; 0.95–1.6). A similar tendency is noted for male gender and mortality, where the HR for participants is 1.8 (1.6–2.1) and for dropouts HR 1.4 (1.1–1.8). Even so, in all cases but one, the CIs overlap, the one exception being the association between a low education level and psychotropic medication, where the HR is 1.1 (1.0–1.1) in participants and 1.3 (1.2–1.5) in dropouts.

## Discussion

Eligibility into the first follow-up of the SPHC was necessarily restricted to the healthy survivors of the period 1999/2000–2005, out of whom men, persons born outside Sweden, persons with poor self-rated overall and mental health and smokers were under-represented. In the present study, it was confirmed the same health determinants, as well as a low education level, were related to dropping out from the follow-up in 2005. Dropping out in 2005 was further associated with a subsequent higher mortality during the 10-year follow-up period; the age-adjusted HR was 1.6 (1.4–1.8).

Thus, there was clearly evidence for self-selection of healthier individuals into participation, which generally entails a risk of underestimating disease occurrence in the underlying population [11, 14, 26]. The age-adjusted HR for psychotropic medication was also slightly higher among dropouts (HR 1.2; 1.1–1.3; Table IIb). It might therefore be reasonable to assume that future population incidence rates of psychiatric conditions inferred from the cohort of participants in this cohort may be too low. Also supporting this assumption is the fact that the general propensity to participate in cohort studies has been reported to be low for persons with psychiatric conditions [26].

The age-adjusted HR for cardiovascular medication did not differ between participants and dropouts. A large part of this medication consists of primary and secondary prevention medication, which demands a willingness to seek and follow medical advice, not only for symptom relief but also for risk reduction. A tendency not to comply with medical advice including prophylactic medication may be linked to the same factors that are associated with non-participation. For instance, it was shown in a recent study that individuals with a larger number of insufficiently controlled cardiovascular risk factors were more likely to be male and have a lower education level [27].

Poor health is often related to socioeconomic status and because socioeconomic status may also influence the willingness to participate, the net influence on participation can be complex. For instance, in a recent study, the prevalence of a history of cardiovascular disease, cancer, or chronic obstructive pulmonary disease was 24% in participants and non-participants alike [28]. This seemingly identical disease risk was tentatively explained by the hypothesis that the low participation of persons with socioeconomic conditions associated with increased risks of disease was balanced by an increased willingness to participate among persons with these diseases.

The second and most important aim of this study was to investigate whether main health determinants had a differential impact on health among participants and dropouts, respectively. We found that patterns of association between the selected health determinants and the outcomes were generally similar in the two groups. This is in line with results from earlier studies, which also indicate that selection is of little importance for the validity of associational effect estimates [10–15, 18]. In a study similar to ours, the authors simulated the influence of non-participation on associations between exposures and outcomes, in that case disability pension, by excluding participants with more symptoms of common mental disorders. This led to a modest reduction of the magnitude of the associations between exposures and risk for disability pensions [14], indicating that selection bias in similar studies, if present, would point in a direction of underestimating associations, rather than the opposite.

In the previous validation study of this cohort at baseline [21], foreign-born individuals were under-represented. In the present study, this flaw was further accentuated, because non-participation at follow-up was strongly related to a non-Swedish origin. In the multivariate analysis, being born outside Sweden was associated with an increased HR of approximately 20% for both mortality and for purchase of prescribed psychotropic drugs among participants, whereas no relationship was seen among dropouts (Table III). The implication of the finding is far from clear, and especially so because the homogeneity of the group of ‘foreign-born’ individuals can be questioned. In a recent systematic review investigating mortality in foreign-born individuals in the Nordic countries, both higher and lower risks were found, depending on the country of origin [29].

In conclusion, our results do not support that loss to follow-up, with self-selection of participants, would invalidate future exposure-outcome results obtained from multivariate analysis to an important degree – at least not regarding the health determinants selected for this study. This is an important finding regarding the SPHC. It also adds to the general knowledge concerning risk for selection bias in population-based cohort studies, even if caution must be made when dropout proportions during follow-up are considerably higher than in the present study.

### Strengths and weaknesses

Important strengths of the study are that it was based on a randomly invited sample from the general population and that a wide range of exposures were available for scrutiny. Another strength is that relevant outcomes covering more than 10 years of follow-up were available by means of registers with an almost complete coverage regarding the cohort members. Moreover, the estimated effects on health outcomes were controlled for confounding by basic demographic factors and daily smoking, self-rated health and mental health, which were measured by well-validated instruments.

A weakness of the study was that only selection bias occurring during follow-up was investigated. It is conceivable that self-selection could have taken place already in the baseline assessment of the invited sample when the SPHC was established in 1999/2000. However, no clear difference in healthcare utilisation costs after age-adjustment among participants and non-participants has been observed at baseline [21]. In contrast, it has been documented that individuals born outside Sweden were under-represented at baseline [21].

## Conclusions

We found evidence for self-selection based on sociodemographic factors, smoking habits and self-assessed health into the first follow-up of the SPHC. Furthermore, subsequent mortality was higher among dropouts. However, the impact of common health determinants on all-cause mortality and on purchase of prescribed cardiovascular and psychotropic drugs during the same follow-up time was generally similar among participants and dropouts. Consequently, there was no support for substantial selection bias in associational measures, which is important information when assessing the validity of prospective findings from this and similar cohorts.
